# In Remembrance of Dr. Mary Ellen Avery

**DOI:** 10.3389/fped.2014.00021

**Published:** 2014-03-21

**Authors:** Joanna Floros

**Affiliations:** ^1^The Pennsylvania State University College of Medicine, Hershey, PA, USA

**Keywords:** remembrance, Mary Ellen Avery, Floros, Neonatology, Echo Lake

Dear Mel,

People the world over know about your academic and medical accomplishments. I would like to share with your colleagues, the Mel that in the 1980s I got to know and spend time with outside the laboratory (although science and how science can benefit “your” babies was just a breath away in anything we did).

The time I really got to talk with Mel outside the Harvard confines was when I first roomed with you in DC, USA. It was a last minute decision to attend the meeting and you so graciously offered to let me room with you. That was a great experience for a young academician to have the exclusive attention of a “giant” like you. The most memorable incident was when you locked me out one evening. What a surprise when the door did not open. Do I risk waking up the “giant” or do I make alternate plans – of course if I didn’t show up all night what would you have thought? You were very kind and apologetic for not thinking when you pulled the lock on. The subsequent evenings I made sure to be the first in the room. Also not to be forgotten on that trip was when you suggested we all stop and play pinball, much to the amusement of some teenagers on a nearby machine.

The trips to “Echo” Lake – what unforgettable memories these were for the entire family. The fish experiment you had planned so carefully. The hypothesis you wanted to test was that application of surfactant (TA surfactant) between the gills of freshly caught and still jumping fish will allow them to breath and survive longer out of the water than their untreated counterparts. Andreas and Nikos (the youngest science helpers you probably ever had) kept track of the timing until the fish stopped kicking. You had them also participate in the actual experiment, holding the fish and opening the gills to apply surfactant while we kept timing. As a good scientist you collected the data. You wrote the manuscript, naming my two young sons, then 8 and 4 years old, as coauthors. The experiment was fun, the hypothesis did not prove correct (if I recall correctly). Unfortunately, this valuable manuscript got lost somewhere between a move to Pennsylvania and a house flood a few years ago.

Not to mention the lobster dinners and the boat rides on Echo Lake with the best captain I ever had who knew all the nesting trees for the ospreys and the best fishing spots. Echo Lake getaways with Mel and family were amazing. It was the first for me to hear the calling of the loons and to see the Aurora Borealis.

Traveling with you whether in Nigeria, Greece, or USA was always a lot of laughs. I miss that! In the midst of laughing and joy you coached me to go where I belonged – to a basic science department. In the ever candid Mel way, you said, “If you stay, we will take advantage of you.” I did move to a basic science department, but now I am back in the Department of Pediatrics. There was something about that early “imprinting” being in Peds. But guess what? It “ain’t” the same without Mel.

Thanks for all you did. You are never far from our thoughts!

Yana

